# Neurocognitive endophenotypes in CGG KI and *Fmr1* KO mouse models of Fragile X-Associated disorders: an analysis of the state of the field

**DOI:** 10.12688/f1000research.2-287.v1

**Published:** 2013-12-27

**Authors:** Michael R. Hunsaker

**Affiliations:** 1 Scientific Computing and Imaging Institute, University of Utah, Salt Lake City, UT, USA

## Abstract

It has become increasingly important that the field of behavioral genetics identifies not only the gross behavioral phenotypes associated with a given mutation, but also the behavioral endophenotypes that scale with the dosage of the particular mutation being studied. Over the past few years, studies evaluating the effects of the polymorphic CGG trinucleotide repeat on the *FMR1* gene underlying Fragile X-Associated Disorders have reported preliminary evidence for a behavioral endophenotype in human Fragile X Premutation carrier populations as well as the CGG knock-in (KI) mouse model. More recently, the behavioral experiments used to test the CGG KI mouse model have been extended to the *Fmr1* knock-out (KO) mouse model. When combined, these data provide compelling evidence for a clear neurocognitive endophenotype in the mouse models of Fragile X-Associated Disorders such that behavioral deficits scale predictably with genetic dosage. Similarly, it appears that the CGG KI mouse effectively models the histopathology in Fragile X-Associated Disorders across CGG repeats well into the full mutation range, resulting in a reliable histopathological endophenotype. These endophenotypes may influence future research directions into treatment strategies for not only Fragile X Syndrome, but also the Fragile X Premutation and Fragile X-Associated Tremor/Ataxia Syndrome (FXTAS).

## Introduction

The *FMR1* gene is polymorphic for the number of CGG trinucleotide repeats on the 5´ untranslated region (5´UTR). In the general population there are fewer than 45 of these CGG trinucleotide repeats. This results in what shall be operationally referred to as *normal* levels of *FMR1* messenger RNA (mRNA), and *normal* levels of the *FMR1* protein (FMRP).

In the Fragile X Premutation, there are between 55–200 CGG repeats (individuals with between 45–55 repeats are referred to as carriers of Grey Zone alleles). In the Fragile X Premutation, there are 2–8 fold increases in *FMR1* mRNA in peripheral leukocytes and reductions in FMRP expression levels that appear to loosely scale with the CGG trinucleotide repeat length ^1– 6^. Carriers of the Fragile X Premutation show increased frequencies of anxiety disorders, neuropsychiatric disorders, and autoimmune as well as other medical co-morbid disorders. Additionally, ~20% of female and ~45% of male Premutation carriers will develop symptoms such as cerebellar gait ataxia, postural sway, intention tremor, Parkinsonism, cognitive decline and dementia, as well as a dysexecutive syndrome during their lifetime. These symptoms have been collectively referred to as Fragile X-Associated Tremor/Ataxia Syndrome (FXTAS). The mechanisms underlying incomplete penetrance of FXTAS in Premutation carriers is an open question currently under investigation ^7– 22^.

In the Fragile X Full Mutation, there are more than 200–230 CGG repeats (often >500 CGG repeats), and in the majority of cases the promoter region of the *FMR1* gene becomes hypermethylated, and expression of the *FMR1* gene is virtually silenced ^4, 23– 26^. This results in a virtual absence of *FMR1* mRNA and almost no measurable FMRP expression. This leads to phenotypes that include intellectual disability, macroorchidism, and autistic-like features collectively known as Fragile X Syndrome ^27– 29^.

Both the Fragile X Premutation and Full Mutation have associated mouse models that have been developed to study them. Specifically, Rob Willemsen and colleagues in Rotterdam developed a CGG knock-in (KI) mouse model (CGG KI) via homologous recombination (in this case replacing the mouse 5´UTR with a 5´UTR containing 99 CGG repeats of human origin) to model the unstable transmission of CGG repeats across generations ^30– 35^. A similar model was developed in 2007 using a CGG-CCG serial ligation method by Karen Usdin and colleagues at NIH (i.e., no human *FMR1* DNA was used ^36^). The CGG KI mouse model recapitulates the neuropathological and somatic pathological features associated with the Fragile X Premutation and FXTAS, namely eosinophilic, ubiquitin immuno-positive intranuclear inclusions bodies in neurons and astroglia in the brain, as well as affecting a range of somatic organ systems and the peripheral autonomic and enteric nervous systems. In 1994, a Dutch consortium developed a mouse model wherein the *Fmr1* gene was knocked out as a model of Fragile X Syndrome ( *Fmr1* KO mouse) ^37^. This mouse recapitulates a number of pathological features of the Fragile X Full Mutation, such as macroorchidism and abnormal dendritic arborization in the brain.

Unfortunately, research using these mouse models of Fragile X-Associated Disorders to elucidate gross behavioral phenotypes has proven at best inconsistent. For each of the separate CGG KI mouse lines it was reported that there were very few behavioral phenotypes, and, even when present, the observed effects were rather small. For the *Fmr1* KO mouse there has been a slightly greater measure of success in identifying behavioral phenotypes, but the presence and magnitude of any observed effects varies widely across labs, behavioral paradigms, and across background strains ^38^.

Based upon these discrepant and counterintuitive findings (i.e., that of inconsistent or absent phenotypes in mice that are clearly not normal), we used the CGG KI mouse developed by Willemsen and colleagues to develop a battery of behavioral tasks that could identify a neurocognitive endophenotype ^38– 46^. We felt that identifying such an endophenotype was possible because the parametric CGG repeat lengths in the CGG KI mouse gave a scalar against which behavioral performance could be associated. Later, an independent group used the paradigms we developed to evaluate behavioral endophenotypes in the *Fmr1* KO mouse model ^47, 48^. This review will describe the process behind developing a behavioral endophenotyping battery as well as unpacking the resulting behavioral profiles from the experimental process and describe how they inform clinical research into Fragile X-Associated Disorders. Emerging results in the quantity and distribution of neuropathological features will be discussed in the context of human disease, although the present murine models do not entirely recapitulate these findings at present.

## Endophenotype approach

There is one clear difference between identifying a behavioral phenotype and identifying a behavioral endophenotype. This difference is that to evaluate a behavioral phenotype, the researcher need only look for a difference in behavior among a homogeneous group of mutant mice relative to a littermate or strain-matched control group. This main effect is then used as evidence for some kind of behavioral impairment. This process is akin to using the same battery of standardized neuropsychological tests to evaluate the behavioral consequences of a number of different genetic disorders and then trying to make inferences about what are the specific profiles of strengths and weaknesses unique to each disorder. In contrast, to evaluate a behavioral endophenotype in the same mice, there is a requirement that any behavioral phenotype predictably scale across some measure. Usually such factors include age, genetic dosage in situations of polymorphic mutations or chromosomal aneuploidy, or some other experimentally controlled factor that is altered parametrically (e.g., stress, environmental toxicant exposure, etc.). This process is similar to the experimental psychology or cognitive neuroscience approaches to studying the behavior of populations carrying genetic mutations. That is, an approach that emphasizes using hypothesis-driven tests that have been designed to evaluate hypothesized effects within the population being studied, irrespective to performance of other populations ^38, 46^.

The importance of finding a behavioral endophenotype is that if there is a predictable relationship among cognitive performance and gene expression, it can be assumed that the genetic mutation alters behavioral output; and subsequently, some sort of relationship between the two exists. Such a finding not only provides a wealth of information that helps the researcher design future experiments, but also data that are useful as outcome measures for studies of intervention that alter or even potentially mitigate some negative impact of the mutation. If there is a more complex relationship wherein age appears to modulate the relationship between the mutation and behavioral output, then those data serve not only as outcome measures, but if well enough understood, could be potentially useful to define risk prodromes to predict future symptomatology or disease progression.

A possible reason for the lack of direct applicability of mouse model research for drug discovery is the unfortunate focus on gross phenotypes that may be either at best secondary to the mutation or result from mouse-unique factors that do not scale evolutionarily. Stated more colloquially, it is much easier to cure disease in mice than to translate the murine research into actually curing human disease. The same general paradigm is prevalent in research into sequelae resultant to neurodevelopmental/neurodegenerative genetic diseases. As a scientific community, we have been able to identify and provide *cures* for a wide range mouse models of genetic disorders (i.e., Fragile X-Associated Disorders), but to date these ‘cures’ have not proven particularly useful for ameliorating symptoms of human genetic disease: often failing or providing only marginal effects during early phase clinical trials ^38^. Elucidating behavioral or neurocognitive endophenotypes using tasks designed to test specific disease-related hypotheses is one proposed solution to mitigate this lack of efficacy in the mouse model ^46^.

For these, as well as many other reasons, research into schizophrenia has forced those in the field to change their general approach, and emphasize an endophenotyping approach in the study of prodromal states associated with schizophrenia onset and symptom progression (e.g., focusing research on longitudinal analyses of 22q11.2 deletion populations rather than on *de novo* schizophrenia cases of unknown or poorly understood genetic origin). By focusing on factors that scale with disease or symptom severity, researchers have been able to understand far more about schizophrenia and what may underlie symptom progression than they would otherwise have been able using a standardized, neuropsychological phenotyping approach ^38, 46^.

## Neurobehavioral endophenotype

### Battery development and implementation

The first step in evaluating the potential for a behavioral phenotype in CGG KI mice was to choose what cognitive domains to test. Unfortunately, at the outset there was a startling lack of data on which to base experimental hypotheses, both from human or mouse research. There were anecdotal data from patients that suggested problems with "foggy thinking" in Premutation carriers, which was interpreted by our clinical collaborators as a combination of abnormal hippocampal and parietal cortex function. A separate collaborator of ours that was interested in neurocognition set out to identify any potential prodromal neurocognitive deficits in Fragile X Premutation carriers, focusing primarily on spatiotemporal processing ^49– 51^. Using both of these pieces of anecdotal evidence in conjunction with the preliminary data from our collaborators as support for our hypothesis, we set out to evaluate the potential for spatial and temporal processing deficits in CGG KI mice. Similarly, we extended our analysis to visuomotor processing as motor learning and motor performance have a strong dependence on intact spatiotemporal integration ^40, 43^.

More to the point, we examined spatial and temporal processing deficits in CGG KI mice using behavioral tasks designed to test specific hypotheses about the cognitive strengths and weaknesses reported in Fragile X Premutation carriers without FXTAS. These cognitive domains were chosen because a number of neurodevelopmental disorders share fundamental spatiotemporal processing deficits described as a *spatiotemporal hypergranularity* ^52^. This hypergranularity, or reduced resolution in temporal and spatial attention, impairs spatiotemporal processing that underlies memory formation. What this means is that a greater difference or separation among elements in space or time is required before they can be discriminated by the research subject.

**Figure 1.**
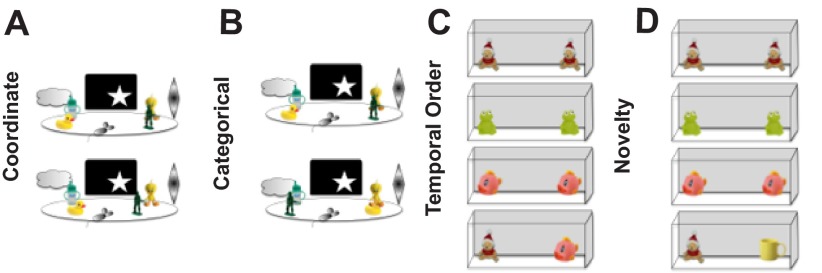
Paradigms used to evaluate spatial and temporal processing in mice. **A**. Metric or Coordinate change detection task. **B**. Topological or Categorical change detection task. **C**. Temporal order for visual objects task. **D**. Novelty detection for visual objects task. All of these paradigms rely on spontaneous exploration to guide behavior, and thus are not confounded by alterations in affect or the requirement to food deprive animals to provide motivation to perform a task to receive a food or water reward.

***Spatial processing.*** Using a pair of behavioral tasks designed specifically to evaluate resolution of spatial processing in CGG KI mice ( Figure 1A-B), it was demonstrated that CGG KI mice show deficits in processing the specific distances that separate two objects in space using a *metric* change detection task (also called a *coordinate* change). These deficits were present in male CGG KI mice as early as 3 months of age, and persisted until at least 12 months of age ^41, 44^.

**Figure 2.**
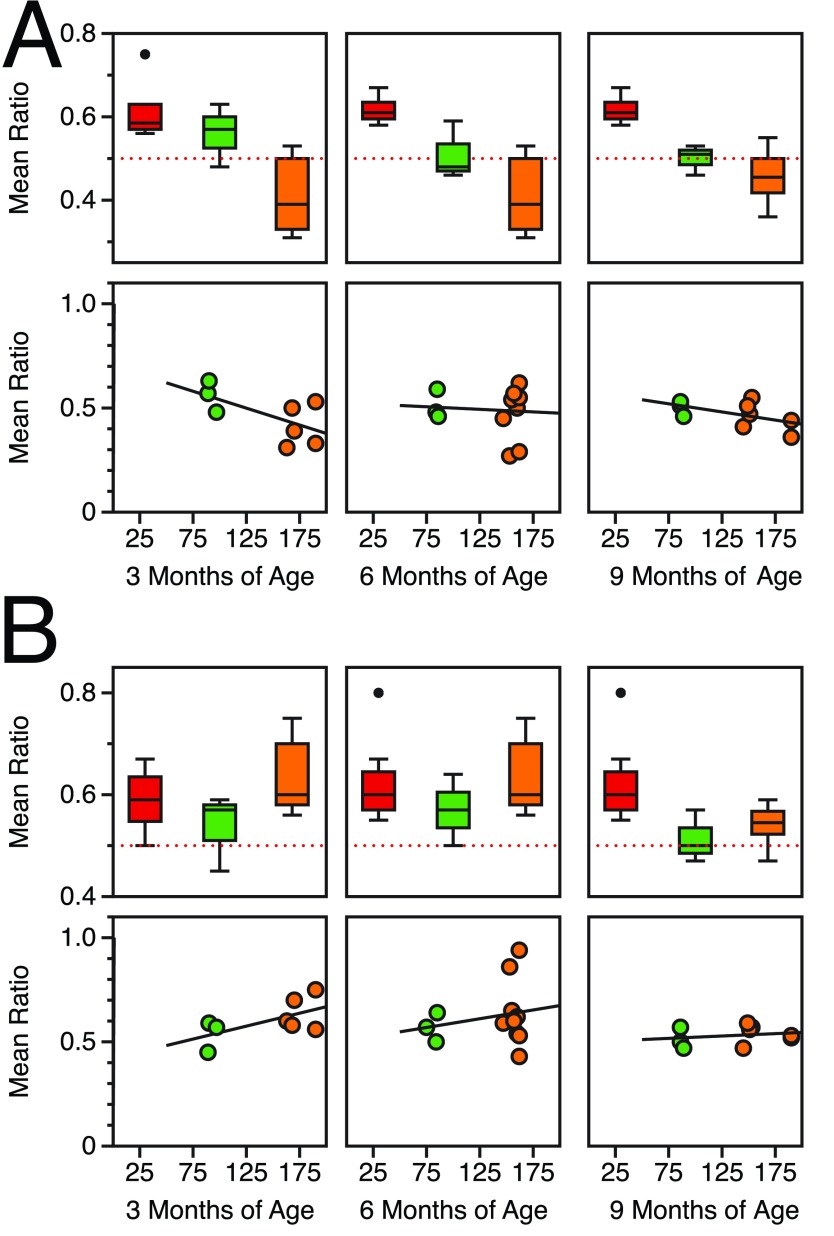
Spatial processing deficits in CGG KI mice scale with CGG repeat length and age. **A**. Metric/Coordinate task by repeat length and age. **B**. Topological/Categorical task by repeat length and age. The red line signifies no response from the animal to spatial change. Data replotted from Hunsaker *et al.*, 2009, 2012 ^41, 44^.

Performance was also tested in a *topological* spatial memory task that required the mice to remember the relative spatial locations of two dissimilar objects after their positions were transposed (also called a *categorical* change). Male CGG KI mice did not show deficits at 3 or 6 months of age, but did show deficits when they were 9 and 12 months of age compared to age-matched wildtype littermate controls ( Figure 2) ^41, 44^.

The results from these spatial experiments suggest that: (1) the resolution of spatial processing in CGG KI mice is reduced at a very young age. This suggests the spatial resolution is due to abnormal development, as this reduced spatial resolution appears to be then be maintained over time, meaning it does not progressively worsen with aging, but *does* show a relationship with CGG repeat length. (2) General spatial memory as measured by the topological change detection task *does* show progressive worsening across age as well as showing a relationship with CGG repeat length, with deficits emerging in adults and becoming more profound with advancing age in CGG KI mice.

**Figure 3.**
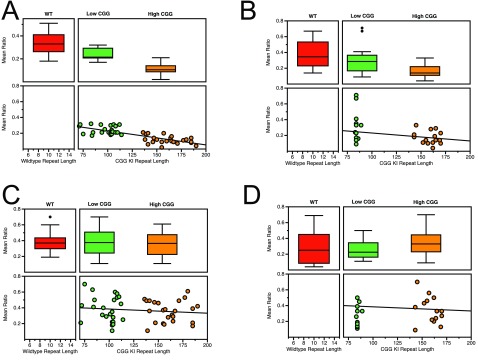
Temporal processing deficits in CGG KI mice scale with CGG repeat length and sex. **A**. Temporal Ordering for Visual Objects Task in male mice. **B**. Temporal Ordering for Visual Objects Task in female mice. **C**. Novelty Detection for Visual Objects Task in male mice. **D**. Novelty Detection for Visual Objects Task in female mice. Data replotted from Hunsaker *et al.*, 2010, 2012 ^42, 44^.

***Temporal processing.*** To evaluate temporal processing in CGG KI mice, a temporal ordering for visual objects task was used. In this task, mice are presented sequentially with three different pairs of identical objects, and time spent exploring the objects is recorded. The mice are then presented with one object from the first pair and one object from the third pair, and time spent exploring is again recorded. Typically, mice preferentially explore the first over the third object. At least 24 hours later to reduce mnemonic interference, after a different set of objects have been presented, the mice are presented with the first object they were presented with that day as well as a never-before-seen novel object. Mice preferentially explore the novel object over the familiar one. On the novelty detection test the CGG KI mice show more exploration of the novel object as do wildtype littermates, but unlike wildtype mice do not show an object preference in the temporal ordering test (i.e., tested with the first and third presented object). This pattern of results indicates that object recognition is intact, but the processing of temporal order is impaired ( Figure 3) ^42, 44^.

An important aspect to these behavioral results is that *both* male *and* female CGG KI mice showed deficits. This is important because female Premutation carriers generally show reduced disease severity due to the protective effect of a second, non-mutated *FMR1* gene on the inactive X allele (i.e., reduced genetic dosage) ^56^. The presence of temporal processing deficits in both male and female CGG KI mice suggests impairments to these processes are fundamental consequences of the Premutation, since deficits are present and identifiable even in the least affected subgroup within the population. Similar effects are currently being identified and characterized in human Premutation carriers both symptomatic and asymptomatic for motor features associated with FXTAS ^49– 51^.

***Motor performance.*** To evaluate potential subclinical gait ataxia or general clumsiness in the CGG KI mice similar to anecdotal reports from our clinical collaborators, a skilled ladder-walking task has been employed. In this test mice are allowed to walk across a series of very thin ladder rungs perpendicular to the direction of travel (similar to walking across a ladder set on the ground). The number of times that the mouse makes an error in foot placement is recorded and is operationally defined as a foot slip. To perform these tasks, the mouse is placed at one end of the apparatus and allowed to cross from one end to the other into a darkened box ^43^.

**Figure 4.**
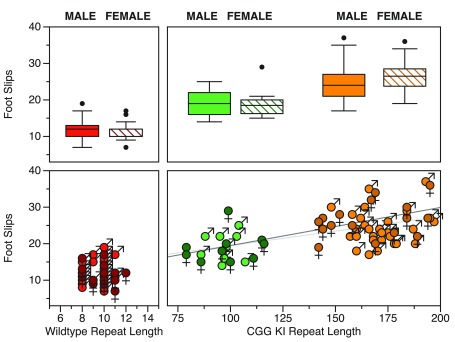
Performance of male and female CGG KI mice on the ladder rung task separated by sex. There were no effects of age present in the data. Data replotted from Hunsaker *et al.*, 2011 ^43^.

The apparatus used in these experiments was modified from previously described ladder rung tasks ^70– 72^. The apparatus had black plastic walls separated by approximately 5cm, with 2mm diameter rungs making up the floor. For this initial study, the mice were placed at one end of the apparatus and were allowed to walk back and forth for 2 minutes. The number of foot slips was recorded for the 2 min duration, except when the mouse was turning around. The number of times the mouse traveled from one end of the apparatus to the other was also recorded as a general locomotor measure. On this task, CGG KI mice as young as 2 months of age already showed an increased number of foot slips compared to wildtype litter mate controls ( Figure 4) ^43^. Importantly, the CGG KI mice showed more of both forelimb and hindlimb slips than wildtype mice, suggesting both visuospatial and basic motor deficits. That is, forelimb foot slips may suggest visuospatial processing deficits, such as difficulty in planning forepaw placement as well as difficulty updating movements as the step progressed (i.e., as the mouse moved forward the initial planned step has to be modified subtly and an inability to do so results in a foot slip). Hind foot slips however, have less of a visuospatial planning component, but may reflect a subtle motor effect. This is because stepping with the hind limbs has been shown to be more efficient and easier for the mouse to perform cf. ^43^. Difficulty in hind limb stepping may therefore reflect some form of ataxia, and may underlie impairments in rotarod and stationary beam walking reported previously ^73^. Similar to the metric or coordinate task data described above, the lack of an age effect suggests that these motor features are due to abnormal development, not an age-related progressive impairment.

### Demonstrating a neurobehavioral endophenotype

***Behavioral analysis.*** After the initial development of the neurocognitive endphenotype described above in the CGG KI mouse model of the Fragile X Premutation, it became clear that these experiments were begging replication in the *Fmr1* KO mouse model of Fragile X Syndrome. We posited two possible outcomes for such a replication and extension study, the first being that the CGG KI mouse and the *Fmr1* KO mouse would show completely independent and non-overlapping deficits wherein CGG repeat dosage would be non-predictive of cognitive deficits. The second suggestion was that the CGG KI mouse and the *Fmr1* KO mouse may show a predictable relationship with increasing CGG repeat length in CGG KI mice and *Fmr1* KO mice showing a linear or otherwise parametric association with each other.

**Figure 5.**
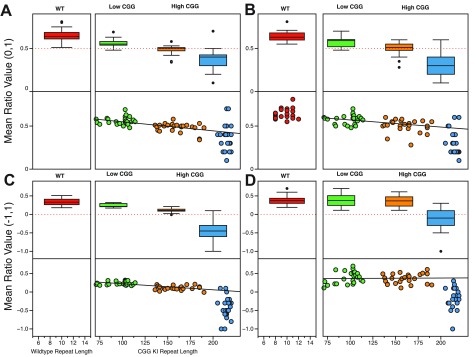
Spatial and temporal processing deficits in CGG KI and *Fmr1* KO mice scale with CGG repeat length. **A**. Metric or Coordinate task. **B**. Topological or Categorical task. **C**. Temporal Ordering for Visual Objects task. **D**. Novelty Detection for Visual Objects task. Data from Hunsaker *et al.*, 2012; King and Jope, 2013; and Franklin *et al.*, 2013 ^44, 47, 48^.

To test these possibilities, we combined our published CGG KI results ^44^ with the data generated by other labs ^47, 48^ wherein they used the battery described above in Figure 1 to evaluate the cognitive deficits in the *Fmr1* KO mouse as part of interventional treatment studies. Overall, the data support both of the proposed outcomes. Figure 5 demonstrates that the CG KI mice and *Fmr1* KO mice show a relatively linear progression in deficits as a function of CGG repeat length toward the Fragile X Full Mutation range for spatial and temporal processing. However, the data also suggest that the *Fmr1* KO mouse demonstrates an unpredicted general memory deficit in the novel object detection task that was not shared with the CGG KI mouse models.

**Figure 6.**
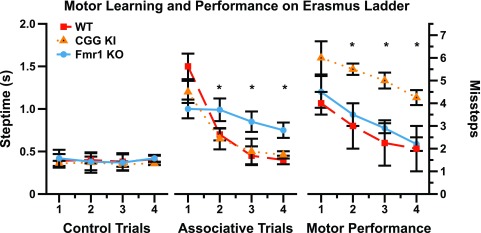
Data redrawn from Cupido (2009) ^53^ demonstrating the dissociation between motor performance and motor associative learning in CGG KI mice and the *Fmr1* KO mouse model of Fragile X Syndrome. Note the dissociation between motor endophenotypes in the CGG KI and *Fmr1* KO Mice. All CGG KI mice were from the High CGG repeat group in other figures.

Using entirely different groups of mice, a group in Rotterdam developed an automated version of the ladder rung task called the Erasmus ladder, upon which the testing and collection of data are fully automated ^53– 55^. Similar to the ladder rung apparatus described above, the Erasmus ladder has a series of horizontal ladder-steps that the mouse must traverse in order to enter an escape/start box at the end. The apparatus was designed to use puffs of air to induce the mouse to shuttle between the two boxes by crossing the ladder steps. The system automatically captures step times, foot slips, misplacement of limbs, etc. Cupido ^53^ found that CGG KI mice showed a significantly increased number of foot slips and missteps ( Figure 6), although they did not differ from wildtype mice in step time, suggesting intact general motor function. Additionally, the CGG KI mice were able to perform the motor associative learning task as well as wild type litter mate control animals. In contrast, *Fmr1* KO mice showed a deficit for the associative learning task, never performing as well as CGG KI and wildtype control animals; however, the number of footslips and missteps did not differ from wildtype controls, suggesting intact visuospatial, visuomotor, and basic motor functions.

It has been demonstrated previously that visuomotor function depends on spatiotemporal integration ^40, 43^. This means that for an individual to act in space, he must be able to (1) select a goal of action and (2) plan the movements, (3) initiate the movements, (4) modify the ongoing movement using updated spatiotemporal information, and (5) properly terminate the movement as intended. Intact spatiotemporal updating prevents misguided movements, such as reaching for a cup and knocking it off the table because an inability to stop or slow the progress of the hand. This is increasingly important if reaching or acting in space extends to processing angles among stimuli or targets that change over time. This updating process is what may be deficient in Fragile X Premutation carriers and in FXTAS patients, and in the CGG KI mice as they show similar spatiotemporal processing deficits and thus reduced spatial and temporal resolution. This type of spatiotemporal deficit may underlie anecdotal reports of subclinical apraxia and general clumsiness among Premutation carriers.

Similar to the novel object detection task, it was clear from the data that the *Fmr1* KO mice demonstrate a deficit in motor learning that was not present in the CGG KI mouse model. The clear dissociation between motor performance (or visuospatial function) deficits in the CGG KI mouse in the absence of motor learning deficits and the inverse case in the *Fmr1* KO mice provides a unique opportunity to evaluate separate components of the motor system in these models. Such data are critical to inform research into the differences between motor phenotypes in Fragile X Premutation carriers with FXTAS and Full Mutation carriers with Fragile X Syndrome.

***Analytical verification.*** As previously demonstrated ^44^, an unsupervised hierarchal clustering algorithm can correctly sort the CGG KI mouse performance on a number of behavioral tasks into appropriate repeat-length subgroups. To verify that these observations comprised a behavioral endophenotype, a similar clustering analysis was performed on a dataset consisting of the CGG KI mice ^44^ and the *Fmr1* KO mice ^47, 48^. Only the metric or coordinate task, the topological or categorical task, the temporal ordering and novelty detection are concluded in this analysis as the number of animals in each group were insufficient in the other experiments reported in the review to run this analysis. In all cases, similar to Hunsaker *et al.* (2012) ^44^, support vector machines (SVM), a supervised machine learning technique, were performed to determine whether patterns in the behavioral data could be interpreted as indications of the expansion of CGG repeat length into the Fragile X Full Mutation. To assess the performance of the SVM classifier, iterative *k-fold* cross validation (10-fold, 5-fold, 3-fold, and leave-one-out cross validation) methods were used to estimate the accuracy of the classifier that predicts the group classification of a test sample. In the figures, the wildtype mice were removed from the dataset and the analyses were repeated to verify that CGG KI and *Fmr1* KO mice could be correctly classified by CGG repeat length (i.e., Low CGG repeat vs. High CGG repeat vs. *Fmr1* KO).

**Figure 7.**
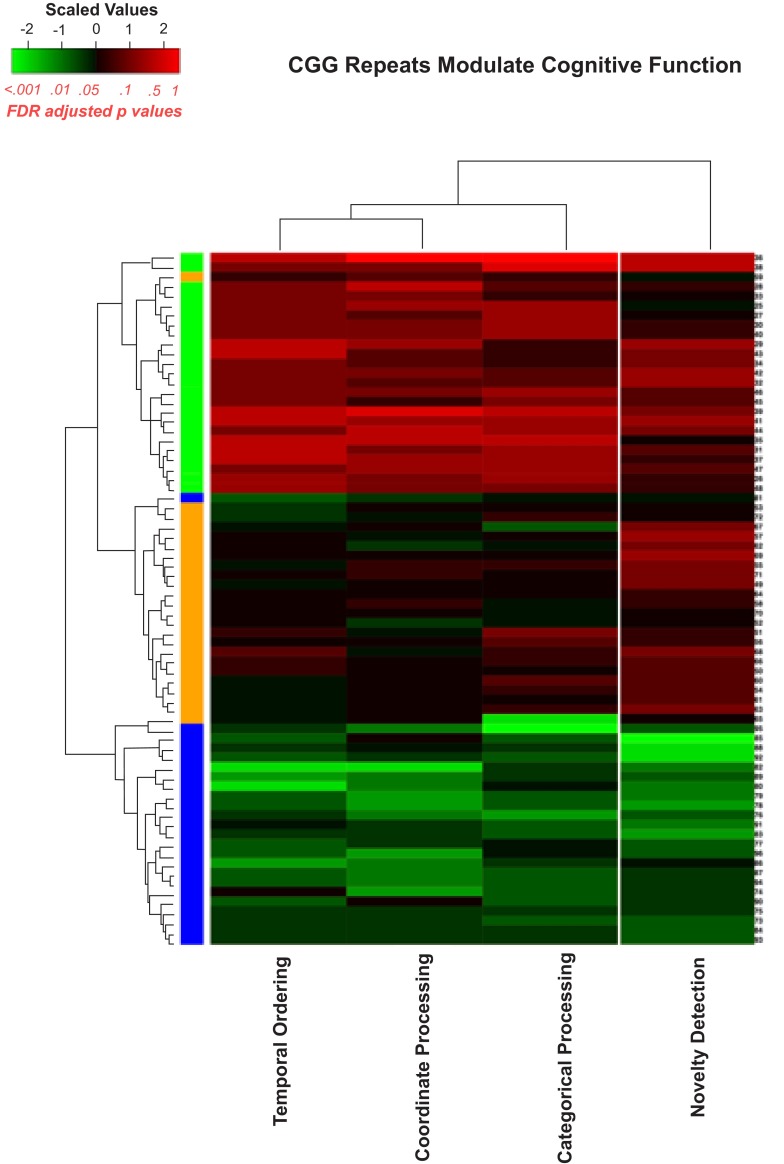
Heatmap visualizing unsupervised hierarchal clustering of CGG KI and *Fmr1* KO mice based on overall behavioral performance. Note how the algorithm was capable of separating groups with high levels of accuracy with only a single *Fmr1* KO mouse and one High CGG KI mouse being misclassified. The colors along the right hand column of the heatmap correspond to the group colors used in Figure 5.

**Figure 8.**
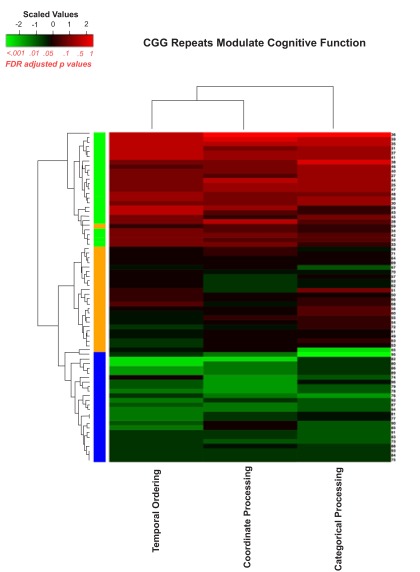
Heatmap visualizing unsupervised hierarchal clustering of CGG KI and *Fmr1* KO mice based on overall behavioral performance. Note how the algorithm was capable of separating groups with high levels of accuracy, with only a single high CGG KI mouse being misclassified. The colors along the right hand column of the heatmap correspond to the group colors used in Figure 5.

Figure 7 is a heatmap representation with all the behavioral tasks included and Figure 8 is a similar heatmap with the novel object detection task removed to evaluate how well the algorithm could sort the *Fmr1* KO mice and CGG KI mice without the benefit of the novel object detection task.

## Emerging histopathological endophenotype

Also emerging from studies evaluating histopathological correlates of the different mutations on the *FMR1* gene are reports that pathologic features are related to CGG repeat length, albeit in a decidedly nonlinear fashion. Table 1 demonstrates this complicated relationship in human Fragile X Premutation and Full Mutation carriers.

**Table 1.** Tabulation of intranuclear inclusion bodies in neurons and astroglia respectively in Fragile X Premutation carrier males and females as well as Full Mutation carrier males and a male that was mosaic for the length of the CGG repeat expansion carrying both Premutation and Full Mutation alleles. Data from Greco *et al.*, 2006 ^62^; Hunsaker *et al.*, 2011 ^43^; Pretto *et al.*, 2013 ^64^; and Tassone *et al.*, 2012 ^20^.

Study	Fragile X-associated disorder	Frontal cortex	Hippocampus-CA1
Greco *et al.*, 2006 ^62^	Male Premutation	4.4%/16.7%	10.1%/10.3%
Tassone *et al.*, 2012 ^20^	Female Premutation	5.6%/4.24%	6.59%/8.84%
Hunsaker *et al.*, 2011 ^43^	Male Full Mutation	0.4%/0.93%	na
Pretto *et al.*, 2013 ^64^	Male size mosaic	3.4%/4.51%	4.09%/4.49%

### Male and female Fragile X Premutation carriers

***Human Premutation carriers.*** Initially, because of the presumed protective effect of a second *FMR1* gene on a non-mutated X chromosome, it was assumed that female Fragile X Premutation carriers would not show similar levels of histopathological features (e.g., intranuclear inclusions) as male Premutation carriers ^56^.

In 2012, a study was undertaken to specifically determine if this assumption was correct ^20^. Specifically, the same methods as employed earlier ^57– 59^ were replicated using sections from female post mortem tissues from Fragile X Premutation carriers. The most intriguing result from this study was that the female Premutation carriers did not in fact have extremely low numbers of intranuclear inclusions in neurons.

The number of intranuclear inclusions in neurons was virtually identical to that observed in male Premutation carrier cases. However, these female cases demonstrated a clear reduction in the number of intranuclear inclusions in astroglia. This reduction, furthermore, seemed to be inversely related to activation ratio, or directly related to the proportion of premutation *FMR1* expressed in brain. This overall effect *does* suggest an overall reduction in histopathological features in female compared to male Premutation carriers, but not a clear cut one ^20^.

An additional datum of interest from this study was that one of the female Premutation carrier cases never developed motor features congruent with a FXTAS diagnosis. In fact, this case was included initially in the sample to demonstrate that the presence of intranuclear inclusions is associated with FXTAS. In other words, it was hypothesized that this case would be either free of inclusions or else have at best very low numbers of these pathologic anatomical features. This case also had a very high activation ratio, meaning the mutated *FMR1* gene was only expressed in 20% of their cells. Despite these presumed protective factors and lack of motor phenotypes during life, this case showed the *highest* number of intranuclear inclusions in the study-suggesting that the inclusion bodies appear to be related to the Fragile X Premutation itself, not to the clinical manifestations of genetic disease (e.g., FXTAS).

***CGG KI mouse model.*** In the CGG KI mouse model a parallel experiment was performed with the female Premutation carrier study described above. What was observed was striking: there was a 20–30% reduction in the number of inclusion bodies in neurons of female CGG KI mice when compared to male CGG KI mice with the same CGG repeat length. For this pathology in astroglia, the female CGG KI mice showed a clear and reliable 50% reduction in intranuclear inclusions compared to the male CGG KI mice with the same CGG repeat length ^60, 61^.

These findings suggest there is a clear dosage effect, at least in mice, for the presence of intranuclear inclusions in CGG KI mice. These data strongly suggest that *Fmr1* expression levels play an as yet undefined role in inclusion body formation.

### Fragile X Premutation and Fragile X Full mutation carriers

***Human Premutation and Full Mutation carriers.*** In 2011, it was discovered that Fragile X Full Mutation carriers counter intuitively show the hallmark neuropathological feature of The Fragile X Premutation and FXTAS: eosinophilic intranuclear inclusions that stain positive for ubiquitin ^62, 63^. To identify how this was possible, given that earlier analyses had suggested that these cases lacked *FMR1* mRNA and FMRP expression, further, more sensitive, molecular analyses were performed. These analyses discovered that *FMR1* mRNA and FMRP were in fact expressed, just at very low levels that were missed by previous techniques ^62, 63^. All said and done, these Fragile X Full Mutation carriers showed approximately 1–2% of the total number of inclusions compared to Fragile X Premutation carriers analyzed in parallel. However, the number of inclusion bodies identified did seem to scale with the rather low expression levels of *FMR1* mRNA and FMRP.

Subsequent studies have borne this hypothesis out by evaluating neuropathologic anatomical feature in individuals that have a size mosaic for the Fragile X Premutation and Full Mutation (i.e., they express alleles with Premutation and alleles with Full Mutation length mutations). In one particular case, the expression of histopathological features again scaled with the expression levels of *FMR1* mRNA and FMRP in brain. Interestingly, this patient had been diagnosed with Fragile X Syndrome and Parkinsonism during their lifetime, but upon the identification of these neuropathologic features and a re-evaluation of the medical records suggested that this individual had both Fragile X Syndrome as well as FXTAS, despite the fact that this comorbidity had previously been discounted as theoretically impossible ^64^.

***CGG KI mouse model.*** In 2012 it was determined that there was a somewhat linear relationship between CGG repeat length and the quantity of intranuclear inclusions in a number of brain areas in the CGG KI mouse ^60^. The similarity with the human cases carrying the Fragile X Full Mutation described above is that mice carrying a mutation upwards of 350 CGG repeats in length were evaluated. Similar to the cases described above, as CGG KI mice show CGG repeat lengths beyond 230 repeats in length, the number of intranuclear inclusions reduces dramatically. This parallels research done in the same mouse line in Rotterdam ^73^ that suggested *Fmr1* mRNA levels and FMRP levels begin to reduce as they approach 200+ CGG repeats.

Unfortunately to date, no matter how long the CGG repeat length becomes in CGG KI mice, the gene does not hypermethylate and silence in the same way as the human *FMR1* gene. This means that at present there is no clear and unambiguous comparison between human and mouse Full Mutation alleles, so far as the analysis of histopathological features associated with disease states is concerned.

## Conclusions

### Behavioral endophenotype

Overall, there are two clear conclusions we can draw from the data described in this review. The first is that the behavioral phenotypes of CGG KI mice and *Fmr1* KO mice appear to be related in some way. This suggests that there is a behavioral spectrum upon which these two mouse models can be found ^38^. Intriguingly, early models positing a spatial and temporal processing deficit model for Fragile X Syndrome and the Fragile X Premutation now have direct experimental support ^65^. We confirmed this by demonstrating that an unsupervised classifier is capable of using the behavioral data from this rapid battery to identify and sort the CGG KI mice into repeat length groups that are distinct from *Fmr1* KO mice, which are reliably sorted as well. These behavioral and analytical results suggest that spatiotemporal processing may be an analytically powerful outcome measure that can be used as an endpoint for interventional, therapeutic, or diagnostic experiments.

However, there is also a clear additional behavioral deficit associated with having the Fragile X Full Mutation that is not present in the Fragile X Premutation. This was best observed in the novel object detection task, wherein the CGG KI mice displayed no deficits whatsoever, whereas the *Fmr1* KO mice showed a rather profound inability to perform the task. The same type of dissociation exists within the motor domain. CGG KI mice appear to show motor performance deficits, possible from impaired visuomotor function but intact motor learning, whereas the *Fmr1* KO mouse shows intact motor function, but impaired motor associative learning. Although these data appear incongruous, the mouse models do actually appropriately model the motor phenotypes associated in the Fragile X-Associated Disorders human population being modeled.

Together the behavioral results reviewed above demonstrating spatiotemporal and motor phenotypes in CGG KI and *Fmr1* KO mice results suggest that the CGG KI and *Fmr1* KO mouse models are valid models for evaluating any behavioral effects of the Fragile X Premutation and Full Mutation. This validity stems from the fact that populations of female Premutation carriers demonstrate similar deficits on spatiotemporal processing tasks as the CGG KI mice and Full Mutation carriers demonstrate clear spatiotemporal deficits on standardized testing. Similarly, motor features in female Fragile X Premutation carriers without FXTAS related motor features have been described. Additionally, research involving Premutation carriers are identifying associations between behavioral performance and CGG repeat length, *FMR1* mRNA expression levels, and FMRP expression levels; further supporting the use of the associated mouse models for preclinical experimentation.

### Histopathological endophenotype

The second main conclusion is that the neuropathological features, although complicated, may also be post-mortem endophenotypes of the Fragile X Premutation and Full Mutation. In humans, it appears that there is a complicated but somewhat predictable relationship between expression of the *FMR1* gene and the presence of intranuclear inclusions, at least within the range of the Fragile X Premutation. In the Full Mutation, it still appears that there is a relationship between *FMR1* expression and pathological features, but this is complicated by the extremely low expression levels and similarly low numbers of intranuclear inclusion bodies ^20, 57– 64, 66– 69^.

In evaluating female and male Fragile X Premutation carriers, it becomes clear that there is an association between gene expression and histopathology-albeit again a complicated one. These results beg for a thorough analysis of cell-type specific *FMR1* expression patterns and cell-type specific analyses of pathology.

Intriguingly, although an incomplete model lacking the *Fmr1* promoter hypermethylation observed in human Fragile X Full Mutation carriers, the CGG KI mouse appears to recapitulate the results of studies carried out with human tissue. Since the CGG KI mouse model appears to model the histopathological features, it is a fair assumption that the CGG KI mouse should be useful to elucidate the process by which these intranuclear inclusions form in different cell types and potentially preclinically test potential therapies or interventions designed to modulate or manipulate the development of this pathology.

### Limitations to the mouse models

Despite the fact that the CGG KI mouse appears to model the Fragile X Premutation relatively well, there are commonly overlooked weaknesses in the mice used to model the Fragile X Full Mutation that need to be overcome. The first of these difficulties is that at present there are not any good mouse models for *FMR1* mutation mosaicism for either size or methylation status. This is important if we are to use the mouse model to adequately model the human condition, given that with increasing study, the genetics underlying Fragile X-Associated Disorders are revealed as more complicated than originally assumed. Additionally, none of the CGG KI mouse models show any evidence for *Fmr1* promoter silencing, so none of the mice show the dramatic reduction in gene expression at the 200–230 CGG repeat threshold often described in human studies.

Although demonstrating similar pathology such as macroorchidism and altered dendritic development, the *Fmr1* KO mouse model does not actually model the genetics of the Fragile X Full Mutation. The *Fmr1* KO mouse was developed by inserting a NEO cassette into the first exon of the *Fmr1* gene, thus silencing expression. However, although apparently a knockout, it was shown that this original *Fmr1* KO mouse *does* express *Fmr1* mRNA and FMRP, albeit nonfunctional and rapidly degraded. This is still the mouse the majority of researchers employ in their research. To eliminate this confound, a second generation of the *Fmr1* KO mouse removed the promoter region entirely, thus completely silencing *Fmr1* gene expression ^74^. The molecular results of these models are very different from hypermethylation virtually, but not absolutely, silencing transcription with the remaining mRNA and protein being normal in structure and completely functional. To fully model the Fragile X Full Mutation at a molecular level, it is necessary for models that show human-like methylation and expression patterns be developed.

### Future directions

Moving forward, the mouse models associated with Fragile X-Associated Disorders (Fragile X Premutation and Full Mutation) will be an essential tool for informing human disease research as well as for preclinical interventional studies. This utility, however, will only occur so long as care is taken to specifically focus on the similarities with the human populations being studied.

For studying the development of neuropathological features, these mouse models may serve as testing platforms for early interventional studies. For the Fragile X Premutation, the CGG KI mouse can be used to determine if there is a critical point in development when an intervention may stave off the development of neuropathological features and potential neurodegeneration (i.e., progression toward a neurodegenerative course associated with FXTAS). Similarly, the *Fmr1* KO mouse is already in use to determine if early intervention can reliably normalize development and dendritic architecture later in life in the absence of chronic treatment, thus improving outcome.

The CGG KI mouse model may further be useful to determine what potential factors contribute to the incomplete penetrance of FXTAS. In other words, one can test environmental toxicant exposure, dietary factors, rearing conditions, anxiety, stress, depression, or any number of factors or combinations of factors to determine if there is an environment × gene dosage × age interaction that underlies abnormal neurodevelopment and eventual neurodegeneration. Studies evaluating potential immune mediated pathology in the Fragile X Premutation are already being evaluated ^75^, particularly for any role of pro inflammatory cytokine levels in long term outcome.

Finally, from a purely behavioral standpoint, it is critical that task development continue for these mouse models that explicitly parallel the tasks being developed for humans and the cognitive and functional domains being emphasized in humans. The current paradigm of murine researchers testing compounds with a focus on prepulse inhibition and the water maze is outdated, not translatable to the clinic in any way, and only serves to stagnate the field. There are currently already a number of resources that provide a solution in the form of behavioral assays that can be creatively applied to model human neuropsychological task batteries ranging from those used currently to test Fragile X-Associated Disorders, the 22q11.2 Deletion Syndrome, and even the NIH Toolbox cognitive assessment tools ( *i.e.*, http://www.nihtoolbox.org). Only by thoroughly modeling the cognitive assays being used can any data using these models be clearly and validly interpreted ^38, 45, 46^.

With any luck, research using the CGG KI and *Fmr1* KO mouse models of Fragile X-Associated Disorders will be able to provide critical information to researchers that can be readily translated to clinical applications. The need for rigorous study of these models is evidenced by the increasing frequency of small pharmaceutical companies developing compounds to treat Fragile X-Associated Disorders. However, to date these efforts have been less than successful in identifying clear hypotheses from the mouse models that can be easily translated into the clinic, much less into clinical trials.

Research into the CGG KI and *Fmr1* KO mouse models and into Fragile X-Associated Disorders are at an impasse that only direct basic-clinical science translational research can overcome. It is only through clear communication across levels of science that the basic scientist and clinician can truly work together toward their common goals. It is only through these reciprocal interactions that innovation truly germinates, and the opportune moment to truly begin is now.
