# Supplementary material for: Neurocognitive endophenotypes in CGG KI and Fmr1 KO mouse models of Fragile X-Associated disorders: an analysis of the state of the field
Source: F1000Res. 2013 Dec 27;2:287. [Version 1] doi: 10.12688/f1000research.2-287.v1 (PMC3945770; doi:10.12688/f1000research.2-287.v1)
Supplement: Peer review history

# Peer review: Referee response for version 1

**Published:** 2014-02-26

**Authors:** Anthony Hannan

**Affiliations:** 1 Behavioural Neuroscience, Florey Institute of Neuroscience and Mental Health, Parkville, Australia

This is a well written review on an important topic. *Fmr1 *knock-out mice have been used as a best approximation of a genetically accurate model of fragile X syndrome, despite the fact that the patients have a tandem repeat expansion rather than a null mutation. The systematic comparison of knock-in and knock-out *Fmr1 *mice is important not just for our understanding of fragile X syndrome, FXTAS and related disorders, but also for the role of this fascinating tandem repeat polymorphism in healthy brain development and cognitive function.  Whilst the maintenance of very long tandem repeat mutations in mouse lines has been a technical challenge (particularly for fragile X syndrome), this can no doubt be overcome so that such tandem repeat disorders are modeled with optimal genetic construct validity. 

The article summarises and integrates a large body of data. The integration of cognitive endophenotypes with information on cellular and molecular mechanisms is informative. I think it is a useful addition to the literature on tandem repeat polymorphisms and associated disorders, and their effects on the healthy and diseased brain.

I have read this submission. I believe that I have an appropriate level of expertise to confirm that it is of an acceptable scientific standard.

# Peer review: Referee response for version 1

**Published:** 2014-02-11

**Authors:** Peter Todd, Abbie Renoux

**Affiliations:** 1 Department of Neurology, University of Michigan, Ann Arbor, MI, USA; 2 Department of Molecular and Integrative Physiology, University of Michigan, Ann Arbor, MI, USA

This review by Michael Hunsaker covers a large body of literature to which he has significantly contributed related to behavioral assay development in Fragile X spectrum disorder mouse models. The author includes data from several different published reports to analyze and compare behavioral results in CGG KI premutation model mice to similar tests done in Fmr1 KO animals. Interestingly, some though not all of these data follow a trajectory related to CGG repeat length.  The author goes on to comment on data comparing inclusion formation in human and mouse models, and address differences between male and female premutation patients and models. It also addresses shortcomings in the current mouse models of Fragile X spectrum disorders. The central conclusion of the paper is that the field needs to work towards development of more patient-applicable endophenotypes to allow for successful translation of basic research discoveries to successful therapeutic development.

 

Overall, the article is well written and the title and abstract are appropriate.

 

Below are a few comments that if addressed would enhance this review and make it more appropriate for indexing.

 

1. **Battery development and implementation: Spatial processing and Figure 1.** **More explanation of these behavioral tasks would aid reader comprehension.  Figure 1 was included to help explain how these tests are performed, but as data are included in the review, it would be useful to have more details on methodology in either the figure legends or in the body of the text. 
   
2. **Figures 2-5.** **A legend explaining that red corresponds to WT, green low CGG, and orange high CGG on these figures should be included either in the figure itself, or in the figure legend.  Additionally, it was not clear that the points outside the box plot were outliers and not indicators of significance. More information about how the data are presented would be helpful. It’s also unclear why the individual data points for the WT results are not always included below the box plots.  Additionally, were any statistics done on these data?  An R ^2 ^value for the correlation lines, or an ANOVA for CGG repeat length, or age, would all be appropriate tests to include in these results without having to refer back to the original research manuscripts.
   
3. **Female premutation model mice and Figures 3 and 4: **The fact that female CGG KI model mice also show differences in temporal processing with these tests is an interesting observation.  Female premutation patients often show less severe symptoms when they have one copy of *FMR1* with smaller repeats, making them heterozygous for the pathogenic repeat allele, owing to X-inactivation and mosaic expression. The genotype of the female mice tested in Figures 3, 4, and the text on temporal processing is not indicated, and should be included for clarity. Sex differences and X-inactivation are also mentioned with the histopathological endophenotype discussion, and warrant more explanation for those unfamiliar with the process and impact that X-inactivation skewing can have on this disorder. Additionally, the result in female mice on temporal processing begs the question if female CGG KI mice have been tested in any of the other behavioral tasks discussed in this manuscript.
   
4. **Figure 5** *:* The author mentions that the Fmr1 KO mice is not a perfect model of FXS as Fmr1 expression was eliminated by inserting a neomycin cassette, and not by way of repeat expansion followed by promoter and 5’UTR hypermethylation as in the human disorder. As such, it is probably incorrect to plot the Fmr1 KO data at/beyond the 200 CGG repeat mark.  These animals do not have any repeats and as such should be plotted on a separate scale.
   
5. **Analytical verification and Figure 7:** Do the two misclassifications noted in Figure 7 correspond to obvious outlier animals?  It might be interesting to look back at the data from those animals which the algorithm did not classify with the rest, and comment on why they may have been missed in the analysis.
   
6. **Histopathological endophenotype:** The author does a nice job of accumulating histological findings in premutation patients and mouse models.  However, the discussion of current hypotheses around the development of inclusion formation and disease pathogenesis is lacking.  There are many theories in the FXTAS field that the increase of *FMR1* mRNA can cause protein sequestration, and may also trigger production of aberrant protein products which could contribute to inclusion formation.  The mechanistic data for both of these hypotheses should be at least mentioned here.
   
7. **Table 1: **The percentages reported for frontal cortex and hippocampus are not explained – is this the percentage of inclusions in neuronal/glial cells?  Additionally, inclusion of CGG repeat lengths where available should be included in this figure, as the author is commenting on the correlation of repeat length and inclusion formation.
   
8. **Conclusions, Behavioral endophenotype: **The comment on associations between CGG length, *FMR1* mRNA, and FMRP expression warrants more discussion.  Illustrating in some way the differential effects of CGG repeat levels on *FMR1* mRNA levels, in contrast to the decrease in FMRP expression with increasing CGG repeats would be helpful (akin to the table generated by ( Brouwer *et al,* 2008).  Further, in discussing these endophenotypes in the context of FXS and the Fmr1 KO mouse, there is a difference between no FMRP expression, reduced FMRP expression, and the toxic mRNA gain of function that are all contributing to the unique phenotypes seen in the fragile X spectrum disorders ( Ludwig *et al.*, 2014; Pretto *et al*., 2014) 
   
9. *Future directions:* The call for more rigorous tests to compare mouse models and human patient phenotypes is indeed warranted.  However, in discussing the limitations of the current models, it should be noted that FXTAS and FXS are not unique in the failings of animal models in general.  The inability of mice to completely recapitulate human neurological disease is very common, especially in neurodegenerative disease (Parkinson’s disease and Alzheimer’s disease models are both notorious for lacking complete recapitulation of clinical phenotypes).  All models (including human iPS cells and simple systems like worms and flies) are fraught with limitations, but there is power in multi-model analysis that one might hope would lead to a greater likelihood for translation from basic science findings to clinical practice.  However, leaving the discussion on therapeutic development as disappointing solely because basic research in mouse models has not yet led to successful clinical trials in patients undermines the significant progress made in the field over the past decade.  

We have read this submission. We believe that we have an appropriate level of expertise to confirm that it is of an acceptable scientific standard.
